# Environmental isolation of *Sporothrix* spp. in veterinary settings

**DOI:** 10.1002/vro2.70019

**Published:** 2025-09-22

**Authors:** Regielly Caroline Raimundo Cognialli, Carolina Melchior do Prado, Giovana Maria da Silva, Tamara Moreira Assad, Ariane Martins, Diego Bejes Sobral, Eelco F. J. Meijer, Vania Aparecida Vicente, Isabella Dib Ferreira Gremião, Flávio de Queiroz‐Telles

**Affiliations:** ^1^ Federal University of Paraná, Hospital de Clínicas Curitiba Brazil; ^2^ Postgraduate Program in Microbiology Parasitology and Pathology Biological Sciences Department of Basic Pathology Federal University of Parana Curitiba Brazil; ^3^ Bolt Veterinary Clinic Curitiba Brazil; ^4^ Department of Medical Microbiology Radboudumc Nijmegen The Netherlands; ^5^ Department of Basic Pathology Federal University of Paraná Curitiba Brazil; ^6^ Laboratory of Clinical Research on Dermatozoonosis in Domestic Animals Evandro Chagas National Institute of Infectious Diseases, Oswaldo Cruz Foundation (Fiocruz) Rio de Janeiro Rio de Janeiro Brazil; ^7^ Department of Public Health Federal University of Paraná Curitiba Brazil

**Keywords:** environmental, infectious diseases, zoonoses

## Abstract

**Background:**

This study investigated environmental contamination by *Sporothrix* spp. in a veterinary facility specialised in treating cats with sporotrichosis.

**Methods:**

Twelve samples were collected from frequently touched surfaces and cages of three cats at different treatment stages. Sampling sites included a procedure table, door handle, faucet, ethanol sprayer, an identification card with visible secretions, and cage components. Samples were cultured on Mycosel agar. Fungal growth was assessed through micromorphological analysis.

**Results:**

*Sporothrix* was isolated from four samples (33%), one from the stainless‐steel procedure table and the others associated with the cages of two symptomatic cats. Cat #1, with active ulcerative lesions, had positive cultures from both the cage grid and latch. Cat #2, in early treatment and showing respiratory signs, had fungal growth on the cage grid. No contamination was detected from Cat #3's environment, and for the remaining sports swapped.

**Conclusion:**

These findings suggest that cage surfaces in veterinary settings can be a source of environmental contamination and potential occupational exposure.

## INTRODUCTION

Sporotrichosis is a neglected mycosis caused by several species of *Sporothrix*, a thermodimorphic fungus; that is, at environmental temperatures of 25°C–27°C it exists in the mycelial phase, while at body temperatures of 35°C–37°C it converts to the yeast phase.^1^ Historically, isolated outbreaks have been reported, with few endemic regions identified in tropical and subtropical areas.^2^, ^3^ These cases were primarily associated with occupational activities and sapronotic transmission, occurring through traumatic implantation of plant‐derived materials contaminated with the mycelial phase of *Sporothrix* species.^2^ However, since the emergence of *Sporothrix brasiliensis* in Brazil in the late 1990s, many aspects of traditional sporotrichosis have changed, particularly transmission routes and clinical manifestations.^4^, ^5^, ^6^ Brazil is currently facing a large outbreak of sporotrichosis by *S. brasiliensis*, which has also spread to other Latin American countries, including Argentina, Paraguay and Chile, as well as imported cases reported in the United States and the UK.^6^, ^7^, ^8^, ^9^, ^10^, ^11^

*S. brasiliensis* is more virulent than other species within the clinical clade (*S. schenckii, S. globosa, S. luriei*), mainly due to melanin production, thermotolerance, and biofilm formation, and it causes a severe disease in cats.^12^, ^13^ Unlike other *Sporothrix* species, which are typically transmitted from the mycelial phase, *S. brasiliensis* can be transmitted directly in its yeast phase.^14^, ^15^, ^16^

In people, sporotrichosis manifests as fixed cutaneous, lymphocutaneous, disseminated cutaneous or extracutaneous forms (e.g., ocular, meningeal, pulmonary or hypersensitivity reactions),^17^ and infections caused by *S. brasiliensis* in these cases are increasingly associated with atypical and extracutaneous manifestations, as well as with more severe and disseminated disease, particularly in immunocompromised individuals.^18^, ^19^ In cats, the disease can present as cutaneous lesions, with the most common clinical presentation being multiple cutaneous lesions with mucosal involvement, which can progress to a disseminated systemic disease.^20^ Domestic cats play a central role in the transmission of *S. brasiliensis* because their lesions carry a high fungal burden, facilitating spread to other cats, dogs and people.^16^ For years, it was believed that infection occurred only through zoonotic transmission, such as bites and scratches from infected cats.^5^ However, recent studies have demonstrated that contact with exudates or sneezes from sick cats on mucous membranes, or even apparently intact skin, can also lead to infection.^21^ Furthermore, recent publications have reported non‐zoonotic transmission of *S. brasiliensis*, including sapronotic routes and possibly through fomites.^16^, ^22^ The possibility of fomite transmission introduces new challenges in the epidemiology of sporotrichosis and raises significant public health concerns. This route of transmission was initially hypothesised in an in vitro study.^16^

This study aimed to evaluate the potential for environmental contamination by *Sporothrix* in a veterinary setting involving cats with sporotrichosis, and to assess the associated risk of fomite transmission.

## MATERIALS AND METHODS

Twelve environmental samples were collected from a veterinary facility exclusively dedicated to the isolation and hospitalisation of cats with sporotrichosis. We selected spots that were frequently touched by the staff members. Samples were collected using a swab moistened with sterile saline and smeared directly onto Mycosel agar (BBL, Becton, Dickinson and Company) for fungal culture. Samples were collected from the following locations: two spots on the stainless‐steel procedure table, one from the door handle, one from the faucet, one from the plastic ethanol sprayer, and one from a cat's identification card, the latter showing visible secretions. Additionally, samples were collected from cage grids and cage latches of three cats selected for their different stages of the treatment scheme.

Fungal cultures were incubated at 30°C, and colonies were evaluated based on macro‐ and micromorphological characteristics. For *Sporothrix* identification, macromorphological analysis revealed membranous colonies ranging in colour from white to beige, occasionally with dark pigmentation. Micromorphological analysis, performed using lactophenol cotton blue staining, demonstrated delicate, branched, septate, hyaline hyphae with sympodially arranged conidiogenesis.^3^

## RESULTS

**FIGURE 1:**
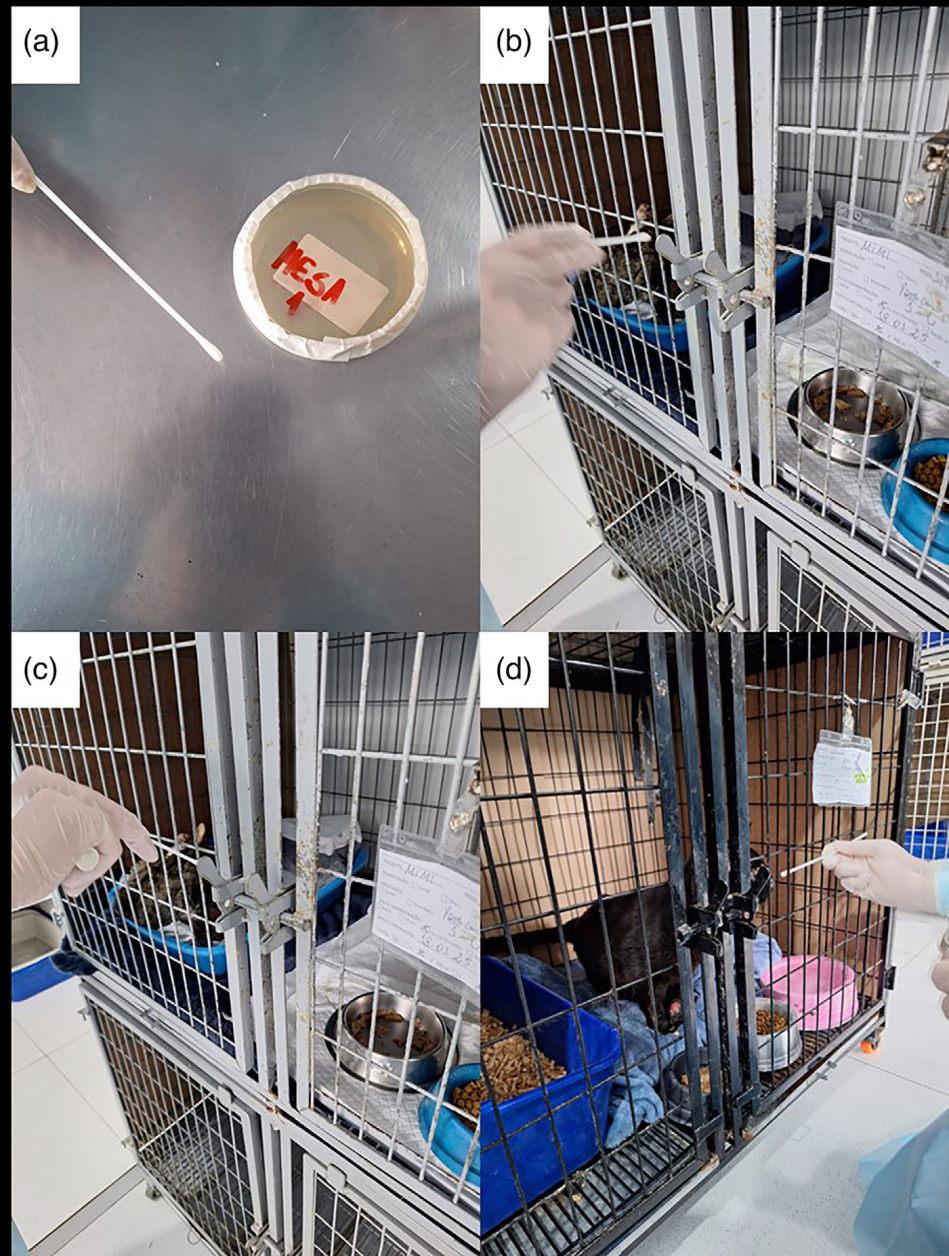
Sites of environmental isolation of *Sporothrix*. (a) Stainless‐steel procedure table; (b) cage grid from Cat #1; (c) cage latch from Cat #1; (d) cage grid from Cat #2.

To assess potential environmental contamination by *Sporothrix*, we collected swab samples from 12 distinct sites (Table 1) within a veterinary clinic housing cats diagnosed with sporotrichosis, and put them on culture. The samples were subsequently cultured, and *Sporothrix* was successfully isolated from four (33%) of them. Remarkably, one positive isolate originated from Site 1 on the stainless‐steel procedure table (Figure 1A), which had appeared visibly clean before sampling. Although molecular methods were not performed for species‐level identification, based on the infection context and the endemicity of *S. brasiliensis* in the veterinary clinical area (Curitiba, Paraná, Brazil),^11^ this species was likely responsible.

**TABLE 1:** Different swapped spots and the culture outcome for *Sporothrix*.

Sampling site	Outcome
Stainless‐steel procedure table Spot 1	Positive
Stainless‐steel procedure table Spot 2	Negative
Door handle	Negative
Faucet	Negative
Plastic ethanol sprayer	Negative
Cat's identification card	Negative
Cage grids Cat #1	Positive
Cage latch Cat #1	Positive
Cage grids Cat #2	Positive
Cage latch Cat #2	Negative
Cage grids Cat #3	Negative
Cage latch Cat #3	Negative

At the time of sampling, cage surfaces from Cats #1 and #2 had positive environmental cultures (Figure 1B–D). For Cat #1, undergoing prolonged treatment and still presenting ulcerated skin lesions, fungal growth was present on both the cage grid and latch. For Cat #2, in the early stage of treatment and exhibiting respiratory signs, a positive culture was obtained from the cage grid only. Cat #3 exhibited only a persistent nasal deformation, and no environmental contamination was found. Table 2 summarises the cats’ clinical status, antifungal treatment and the corresponding positive environmental samples.

**TABLE 2:** Clinical characteristics, antifungal treatment and environmental isolation of *Sporothrix* from cages.

Cat ID	Treatment duration (months)	Current clinical presentation	Antifungal treatment	Positive environmental sample(s)
Cat #1	14	Multiple ulcerated skin lesions	ITZ + KI (10 months)/POS	Cage grid, cage latch
Cat #2	4	Respiratory signs (sneezing)	POS	Cage grid
Cat #3	12	Nasal deformation	ITZ + KI (7 months)/POS	None

Abbreviations: ITZ, itraconazole; KI, potassium iodide; POS, posaconazole.

## DISCUSSION

The findings of this study provide real‐world evidence of *Sporothrix* persistence on environmental surfaces, particularly stainless steel. The successful fungal recovery in culture confirms the viability of *Sporothrix* on these materials, underscoring the tangible risk of fomite‐mediated transmission.

Our study successfully isolated *Sporothrix* from four environmental samples, an outcome that contrasts with previous findings. For instance, a study conducted in a hyperendemic area of Rio de Janeiro failed to isolate *Sporothrix* from any of the 18 soil samples analysed.^23^ Similarly, Lara da Acosta et al.^24^ collected soil samples from the homes of cats diagnosed with sporotrichosis, but were also unable to recover the fungi. In contrast, another investigation managed to isolate *S. brasiliensis* from a wood sample collected from a household with a history of a feline sporotrichosis case 3 years earlier.^25^ Additionally, a separate study reported the isolation of *S. brasiliensis* from one of two faecal samples collected from a sand heap in the backyard of a house with infected cats, and also identified *Sporothrix* in two of 24 environmental samples from a metropolitan area in São Paulo, where feline sporotrichosis had been reported.^26^ It is also important to emphasise that while most environmental studies have focused on sampling soil in areas with known cases, to the best of our knowledge, our study is the first to address the issue of potential contamination within veterinary settings.

Environmental isolation of *Sporothrix* remains challenging, largely due to the absence of standardised methodologies specifically designed for this purpose. Regarding sampling methodology, there are different methods for detecting fungi, such as the International Organization for Standardization for surface sampling.^27^ However, as we collected from irregular surfaces and our primary objective was to determine the presence or absence of *Sporothrix* spp. and not quantify the fungal burden, we opted for a more flexible approach using swabs. Future environmental studies should aim to standardise sampling and detection protocols, ideally incorporating metagenomic approaches.^28^, ^29^ Applying such methods in both hyperendemic areas and in regions without reported cases could be key to answering the unresolved question of how *Sporothrix* persists in the environment.

Although itraconazole is considered effective in reducing fungal burden in lesions of cats with sporotrichosis,^30^ our study demonstrated environmental isolation of *Sporothrix* spp. from cages of cats receiving itraconazole therapy for over 4 months. This can be explained by several factors that influence the persistence of fungal burden and potential for environmental contamination. First, cats with disseminated or severe clinical forms tend to exhibit a higher fungal burden and prolonged time to clinical cure.^30^ Second, genetic differences among *Sporothrix* isolates, particularly in virulence and pathogenicity, may affect treatment response and fungal persistence.^31^ Lastly, the effectiveness of itraconazole treatment can be influenced by pharmacokinetic and pharmacodynamic aspects, including drug absorption, dosage, formulation stability and route of administration.^32^, ^33^, ^34^ Compounded formulations, often used in veterinary settings, may exhibit reduced bioavailability and variable efficacy, potentially contributing to subtherapeutic drug levels and continued fungal shedding.^35^

Veterinary clinics should be a primary focus of investigations regarding surface contamination with *Sporothrix*. These facilities may present a heightened risk of fungal transmission, particularly to the professionals working in these environments. Understanding contamination dynamics in clinical settings is, therefore, critical for both occupational health and broader public health control strategies. Cats with multiple lesions and high fungal burdens tend to have persistent lesions and a higher risk of treatment failure.^36^, ^37^ Despite a prolonged period of antifungal treatment, Cat #1 presented a poor clinical response with multiple ulcerated skin lesions, which could have increased the likelihood of surface contamination with *Sporothrix*. Another important condition is the presence of respiratory symptoms, such as sneezing, as seen in Cat #2, which can release respiratory droplets over a distance and contaminate the environment. We did not isolate viable *Sporothrix* from the cage of Cat #3, which is probably a direct consequence of the absence of ulcerated skin lesions and sneezing. For treated cats without clinical symptoms—such as ulcerated lesions or respiratory signs—environmental contamination is likely minimal, but should still be considered. Successful treatment leading to clinical improvement may significantly reduce the risk of environmental contamination and subsequent transmission to other susceptible hosts.

In domestic or veterinary facilities housing cats with sporotrichosis in isolation, environmental contamination with *Sporothrix* is expected. To minimise transmission risks, access to these areas should be restricted to essential personnel only. Strict infection control measures must be implemented, including the use of appropriate personal protective equipment during animal handling, wearing gloves when contacting surfaces, and avoiding hand‐to‐mucous membrane exposure. Furthermore, these areas require routine cleaning with effective disinfectants, such as sodium hypochlorite, to reduce fomite‐mediated transmission.^16^, ^38^

Overall, these findings support the importance of strict hygiene and biosafety measures in the management of feline sporotrichosis, particularly in cases involving severe clinical manifestations or coinfections that may impair treatment response. The potential for indirect transmission via contaminated surfaces or droplets highlights the need for comprehensive infection control strategies in veterinary clinical settings.

## AUTHOR CONTRIBUTIONS

**Regielly C. R. Cognialli** and **Carolina M. do Prado**: Investigation; methodology, visualisation, formal analysis, data curation, writing—original draft, writing—review and editing. **Giovana M. Silva**, **Tamara M. Assad**, **Ariane Martins**, **Diego B. Sobral**: Investigation, writing—review and editing. **Vania A. Vicente**: Writing—review and editing. **Isabella D. F. Gremião**: writing—original draft, writing—review and editing; **Flávio Queiroz‐Telles**: Writing—review and editing.

## CONFLICTS OF INTEREST

The authors declare they have no conflicts of interest.

## ETHICS STATEMENT

This study was approved by the HC/UFPR ethical committee (registration no. 12379819.4.0000.0096).

## Data Availability

Data sharing is not applicable to this article as no datasets were generated or analysed during the current study.
